# The Association of Early Life Supplemental Nutrition With Lean Body Mass and Grip Strength in Adulthood: Evidence From APCAPS

**DOI:** 10.1093/aje/kwt332

**Published:** 2014-02-19

**Authors:** Bharati Kulkarni, Hannah Kuper, K. V. Radhakrishna, Andrew P. Hills, Nuala M. Byrne, Amy Taylor, Ruth Sullivan, Liza Bowen, Jonathan C. Wells, Yoav Ben-Shlomo, George Davey Smith, Shah Ebrahim, Sanjay Kinra

**Keywords:** body composition, cohort study, developmental origins of health and disease, grip strength, lean body mass, muscle mass, nutrition, physical activity

## Abstract

In the present study, we examined the associations of early nutrition with adult lean body mass (LBM) and muscle strength in a birth cohort that was established to assess the long-term impact of a nutrition program. Participants (*n* = 1,446, 32% female) were born near Hyderabad, India, in 29 villages from 1987 to 1990, during which time only intervention villages (*n* = 15) had a government program that offered balanced protein-calorie supplementation to pregnant women and children. Participants’ LBM and appendicular skeletal muscle mass were measured using dual energy x-ray absorptiometry; grip strength and information on lifestyle indicators, including diet and physical activity level, were also obtained. Ages (mean = 20.3 years) and body mass indexes (weight (kg)/height (m)^2^; mean = 19.5) of participants in 2 groups were similar. Current dietary energy intake was higher in the intervention group. Unadjusted LBM and grip strength were similar in 2 groups. After adjustment for potential confounders, the intervention group had lower LBM (β = −0.75; *P* = 0.03), appendicular skeletal muscle mass, and grip strength than did controls, but these differences were small in magnitude (<0.1 standard deviation). Multivariable regression analyses showed that current socioeconomic position, energy intake, and physical activity level had a positive association with adult LBM and muscle strength. This study could not detect a “programming” effect of early nutrition supplementation on adult LBM and muscle strength.

Muscle mass, a major component of lean body mass (LBM), is important for insulin-stimulated plasma glucose uptake and has an independent association with insulin sensitivity (1, 2). Functional competence of muscle tissue (assessed by measuring grip strength) is indicative of improved general health and is associated with a decreased risk of chronic diseases and premature death (3). Moreover, LBM and muscle strength are important measures of human capital, and understanding their determinants is important.

Evidence suggests that early-life nutrition may “program” LBM and muscle strength. Low birth weight was associated with lower LBM and grip strength during adulthood in observational studies from the United Kingdom, Finland, and India (4–7). A major criticism of these studies is the use of birth weight, which may be a poor measure of intrauterine nutrition because birth weight is also influenced by non-nutritional factors (8). A few studies that examined the association between early nutrition intervention and adult body composition in follow-up studies of nutrition supplementation trials in pregnant women provided more direct evidence of the influence of early nutrition exposure, but their results were inconsistent. A study from Guatemala showed a positive association between maternal nutrition supplementation and offspring LBM (9), whereas another study from Gambia did not find such association (10). However, these studies that assessed the influence of nutrition supplementation in the controlled setting of randomized controlled trials did not provide information on any potential long-term effect of these interventions when provided through publicly funded programs.

We therefore examined the predictors of adult LBM and muscle strength in young adults born within a community trial of protein-energy supplementation that was created to examine the impact of a government food supplementation program on birth outcomes in pregnant women. We hypothesized that early nutrition would be an important predictor of LBM and its functional competence in these rural young adults and that participants in the intervention group would have higher LBM and muscle strength than would controls. Given that current lifestyle, including dietary protein intake and physical activity level, can have a significant impact on the muscle mass and LBM (11–13), we additionally examined the role of current lifestyle factors because of their potential to influence the outcome.

## METHODS

### Study design

#### Initial trial (1987–1990) and first follow-up study (2003–2005)

The present study represents a second follow-up of the Andhra Pradesh Children and Parents Study (APCAPS) birth cohort, which was established to assess the long-term impact of nutrition supplementation provided through a government program. The cohort profile and details of the initial trial and the first follow-up study have been reported previously (14, 15). In brief, the initial trial was conducted in 29 villages near Hyderabad, India (1987–1990), using an opportunity afforded by stepwise expansion of a nutritional supplementation program (Integrated Child Development Services scheme). In intervention villages (*n* = 15) only, a nutritional supplement (a freshly cooked preparation made of corn–soya blend and soybean oil) was available daily to all pregnant and lactating women and children less than 6 years of age, providing on average 2.09 MJ of energy and 20–25 g of protein to women and 1.25 MJ of energy and 8–10 g of protein to children. Women had to collect the supplement daily from the program center, but they were not obliged to eat it there. Although precise rates of adherence to the supplementation were not available, a high intake was likely, as considerable efforts were made during the original trial to ensure consumption of the supplement (14). The supplementation was associated with a small but statistically robust increase (61 g, 95% confidence interval: 18, 104; *P* = 0.007) in birth weight of the offspring (*n* = 2,601). The first follow-up study included 1,165 of the children (then 13–18 years of age) and examined the prevalence of risk factors for cardiovascular disease in relation to the intervention. Adolescents from the intervention villages were 14 mm (95% confidence interval: 4, 23; *P* = 0.007) taller than controls and had favorable cardiovascular risk profiles but similar body compositions (15).

#### Second follow-up study (2009–2010)

Between January 2009 and December 2010, we invited potential participants for a second follow-up study to assess markers of chronic diseases. Results for other outcomes will be published separately. Individuals born in the study villages during the period of initial trial, that is, 1987–1990, were eligible for inclusion in the study irrespective of the availability of their birth-weight record from the initial study to avoid selection bias.

Ethical approval for the study was obtained from the ethics committees of National Institute of Nutrition, Hyderabad, India; the London School of Hygiene and Tropical Medicine, United Kingdom; and the Queensland University of Technology, Australia. Approval was also sought from the local authorities. Written informed consent (or witnessed thumbprint if illiterate) was obtained from all the participants.

### Measurements

Consenting participants visited a clinic at the National Institute of Nutrition, Hyderabad. A structured questionnaire was administered to all participants by a trained interviewer to assess background information. Socioeconomic position was examined using standard of living index, which is a household-level asset-based scale devised for Indian surveys (16). This index has been widely used in epidemiologic studies from India (17) and was found to correlate highly with income data (18). Dietary intakes over the past year were estimated using a validated food frequency questionnaire that assessed the frequency of intake of 98 commonly consumed food items (19). Indian food composition tables were then used to estimate the nutrient content of a single portion of each food item (20). Physical activity during the previous month was assessed using a validated questionnaire (21) across the following activity categories: work, travel, sports/games/exercise, household, and sedentary. Information was collected on the frequency and duration of each activity. Metabolic equivalents of tasks (METs) were then calculated as the multiples of resting metabolic rate (1 MET is equivalent to the energy expenditure value of sitting quietly) using the Compendium of Physical Activity and World Health Organization/Food and Agriculture Organization/United Nations guidelines, supplemented with country specific values. Total activity was calculated as total METs (hour/day) by summing daily MET values of all activities (22, 23).

Weight was measured (to the nearest 0.1 kg) using a digital SECA balance (Hamburg, Germany), and standing height was measured (to the nearest 1 mm) with a stadiometer (Leicester height measure, Chasmors Ltd., London, United Kingdom). Each measure was assessed twice, and the average of the 2 values was used in the analysis. Body mass index was calculated by dividing weight in kilograms by height in meters squared. Grip strength was measured separately for each arm using a grip dynamometer (Grip D, Takei, Tokyo, Japan), and the value from the dominant arm was used in the analysis. This measurement was done in the morning after breakfast 3 times, and the maximal estimate of the force was recorded.

LBM was assessed with dual energy x-ray absorptiometry (DXA) (using either a Hologic Discovery A model (91% of scans) or a Hologic 4500W (9% of scans) (Hologic, Waltham, Massachusetts)). The scanner was calibrated daily using a phantom supplied by the manufacturer, and its performance was monitored as per the quality assurance protocol. No sign of scanner drift was observed during the study period. The in vivo precision (coefficient of variation) was less than 1% for the LBM measurement. Standard Hologic software options were used to define regions of the body (head, arms, trunk, and legs). Appendicular skeletal muscle mass (ASM) was calculated as the sum of bone-free lean tissue measurements in arms and legs (24). Pregnant women were excluded from the DXA scanning.

### Quality control

We produced detailed protocols and used them regularly to standardize work of the fieldwork team. Masking the group assignment from fieldworkers was not an option, but the key outcome measures (LBM and ASM assessed by DXA) were automated, which reduced the possibility of bias. Grip strength measurements were undertaken by 2 observers, and the interobserver bias was estimated periodically. Reproducibility of grip strength measurements assessed in a subsample showed high reliability (intraclass correlation coefficient, 0.95). DXA scans were analyzed by a trained technician. Incomplete scans or those with major movement artifacts were excluded from the analyses. Sensitivity analyses showed that their exclusion did not make a difference to the results.

### Statistical analyses

Analyses were conducted using Stata, version 11.2 (StataCorp LP, College Station, Texas). All *P* values were 2-sided. For dietary energy and protein intake measurements, extreme values (those less than the 1st percentile and those greater than the 99th percentile) were adjusted and made equivalent to the values of 1st and 99th percentiles, respectively. Protein intake was examined using the nutrient residual energy adjustment method, which provides a measure of protein intake that is independent of total energy intake (25). Differences in participant characteristics in relation to the supplemental nutrition were assessed separately for men and women using a Student's *t* test for continuous variables and a χ^2^ test for trend for categorical variables, with appropriate degrees of freedom. Unadjusted associations between supplemental nutrition and outcome variables (LBM, ASM, and grip strength) were assessed using linear regression models with robust standard errors to account for clustering by village and sibling pairs. To examine the predictors of LBM, ASM, and grip strength, linear regression models were used with each of these outcomes as a dependent variable and the physiological (age and sex), socioeconomic (educational level, occupation, and household standard of living index), and lifestyle (dietary intakes and physical activity level) indicators as independent predictor variables. Continuous predictor variables (standard of living index, physical activity level, and dietary energy and protein intakes) were divided into tertiles because of their nonlinear relationship with the outcome variables and the known imprecision in these estimates. Tests for linear trends across tertiles of these predictors were conducted using the median value in each tertile as a continuous variable in the linear regression models. These models were additionally adjusted for early nutrition supplementation. Finally, to examine the association between supplemental nutrition and the outcome variables, multiple linear regression models were constructed after adjustment for the potential confounders described earlier. Two predefined models were fitted to adjust incrementally for the main domains of potential confounding or intermediary variables mentioned above: Model 1 was adjusted for physiological variables and model 2 was adjusted for socioeconomic and lifestyle indicators. As height was related to all of the lean mass indicators (for LBM, *R* = 0.87; for ASM, *R* = 0.84; for grip strength, *R* = 0.70; all *P* < 0.001), an additional model (model 3) that included height along with the variables included in model 2 was constructed to assess the extent to which the variation in lean mass indicators in relation to the confounders is mediated by change in height. Estimates of LBM and ASM in all of the above analyses were additionally adjusted for the DXA scanner used. We pooled the sexes for the multiple regression analyses, as there was no evidence of an interaction between nutrition intervention and sex. Robust standard errors were used throughout to account for clustering of the data by village and sibling pairs. Examination of residuals after fitting the regression models for the main outcome variables showed a normal distribution, eliminating the possibility of bias. Missing data were handled with list-wise deletion. Sample-size calculations undertaken before the study commenced suggested that the anticipated sample (about 1,400) would provide adequate power to detect relatively small differences (about 0.17 of a standard deviation) in total LBM, ASM, and grip strength with 90% power and 5% level of significance.

## RESULTS

**Figure 1.**
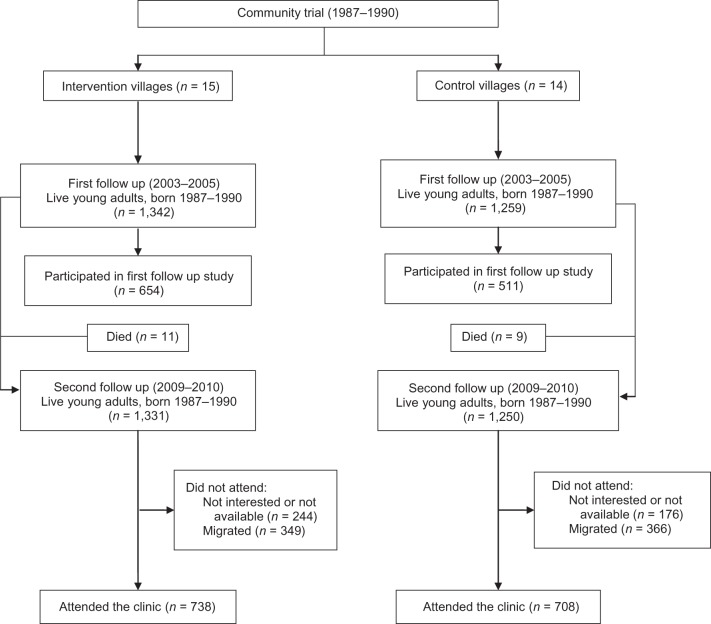
Flow chart of participant recruitment in the Andhra Pradesh Children and Parents Study, Hyderabad, India, 2009–2010.

Of the 2,601 children eligible for inclusion in the study (i.e., those born in these villages during 1987–1990), a total of 1,446 individuals (32% women) participated in the second follow-up study. There were 738 in the intervention area and 708 in the control area, representing a response rate of 56% (Figure 1). There were no major differences in the sociodemographic characteristics of participants and eligible nonparticipants in both the arms of the study (Appendix Table 1). However, a larger proportion of women were lost to follow-up (because of their migration out of the study area consequent to marriage), and participants were more likely to be students than were nonparticipants. LBM and ASM estimates by DXA were available for 1,384 (96%) participants, with data missing for 49 women and 13 men. The main reason for missing DXA data in women was pregnancy; in men, exclusion of DXA scans was typically because of poor quality.

Table 1 shows participant characteristics and the distribution of key exposures in the intervention and control groups of men and women. Although ages and physical activity levels were similar in the 2 groups, dietary energy and protein intakes (in men only) were higher in intervention group than in the control group. Overall dietary intakes were higher than those reported by nutritional surveys in India (especially in men) (26), probably because of overestimation by the food frequency questionnaire, which has been observed in other studies (19, 27). The majority of the men were either students or were engaged in manual occupations, whereas majority of the women were homemakers. Higher proportions of men and women from the intervention group had received higher education than the control men and women, and a larger proportion of control women were engaged in manual occupations (33.9% vs. 25.8%; *P* = 0.04). Socioeconomic position was, however, not different in the 2 groups of participants. Overall, the participants were of relatively short stature with a low body mass index (for men, mean = 19.7; for women, mean = 19.1). The heights, weights, and body mass indexes were not different in the 2 groups of participants.

**Table 1.** Characteristics of Participants Included in the Follow-up Study of the Andhra Pradesh Children and Parents Study Birth Cohort, Hyderabad, India, 2009–2010

Variable	Men					Women				
	Intervention (*n* = 499)		Control (*n* = 482)		*P* Value	Intervention (*n* = 239)		Control (*n* = 226)		*P* Value^a^
	Mean (SD)	%	Mean (SD)	%		Mean (SD)	%	Mean (SD)	%	
Age, years	20.8 (1.1)		20.8 (1.2)		0.37	21.0 (1.1)		21.1 (1.2)		0.33
Dietary intakes										
Energy intake, kcal/day	3,366 (1,146)		3,186 (1,020)		<0.01	2,149 (690)		2,024 (547)		0.03
Protein intake, g/day	82 (30)		78 (27)		0.03	51 (17)		49 (15)		0.17
Protein intake per kg of body weight	1.5 (0.5)		1.4 (0.5)			1.2 (0.4)		1.1 (0.3)		
Physical activity										
Physical activity, MET-hour/day	40.2 (6.4)		40.0 (6.7)		0.80	36.6 (5.3)		36.8 (5.4)		0.66
Time spent in MVPA, minute/day	220 (135)		223 (151)		0.76	118 (123)		104 (122)		0.22
Occupation										
No paid employment^b^		44.1		42.1	0.30		71.7		62.6	0.04
Manual work (skilled/unskilled)		47.9		51.9			25.8		33.9	
Professional		8.0		6.0			2.5		3.5	
Educational level										
≤Primary school		15.4		19.5	0.01		19.6		37.4	<0.01
Secondary school		76.7		76.6			71.2		59.5	
College		7.8		3.9			9.2		3.1	
Standard of living index^c^	18.7 (4.2)		18.6 (4.2)		0.57	17.9 (4.2)		17.2 (4.7)		0.09
Anthropometry										
Height, cm	166.6 (6.4)		166.7 (6.0)		0.63	152.7 (5.1)		152.5 (5.4)		0.69
Weight, kg	54.8 (8.8)		54.9 (8.6)		0.88	44.2 (7.8)		45.0 (7.2)		0.23
Body mass index^d^	19.7 (2.8)		19.7 (2.8)		0.92	18.9 (3.1)		19.3 (2.7)		0.15

Abbreviations: MET-hour/day, metabolic equivalents of tasks (hour/day); MVPA, moderate and vigorous intensity physical activity.

^a^ *P* values were based on unpaired *t* tests or χ^2^ tests for trend. All *P* values were 2-sided.

^b^ This category included homemakers, students, and the unemployed.

^c^ Higher values indicate higher socioeconomic position.

^d^ Weight (kg)/height (m)^2^.

Table 2 shows the distribution of outcome variables in men and women from the 2 groups. LBM and grip strength were largely similar in 2 groups, but ASM was lower in men in the intervention group (mean = 19.78 (standard deviation, 2.71)kg) than in men in the control group (mean = 20.24 (standard deviation, 2.67) kg) (*P* = 0.03).

**Table 2.** Distribution of Outcome Variables in the Intervention and Control Groups of Participants Included in the Follow-up Study of Andhra Pradesh Children and Parents Study Birth Cohort, Hyderabad, India, 2009–2010

Variable	Men					Women				
	Intervention (*n* = 490), mean (SD)	Control (*n* = 478) , mean (SD)	Difference	95% CI	*P* Value	Intervention (*n* = 217), mean (SD)	Control (*n* = 199), mean (SD)	Difference	95% CI	*P* Value^a^
LBM, kg	43.03 (5.49)	43.78 (5.37)	−0.74	−1.58, 0.10	0.08	29.60 (3.87)	30.20 (3.95)	−0.67	−1.5, 0.19	0.13
ASM, kg	19.78 (2.71)	20.24 (2.67)	−0.45	−0.86, −0.04	0.03	12.56 (1.81)	12.87 (1.91)	−0.34	−0.79, 0.10	0.13
Grip strength^b^, kg	33.11 (6.24)	33.65 (5.78)	−0.59	−1.44, 0.27	0.18	20.60 (3.97)	21.10 (3.68)	−0.39	−1.09, 0.31	0.27

Abbreviations: ASM, appendicular skeletal muscle mass; CI, confidence interval; LBM, lean body mass; SD, standard deviation.

^a^ *P* values (2-sided) were based on linear regression models with robust standard errors to account for clustering by village and sibling pair. Dual energy x-ray absorptiometry estimates of LBM and ASM were additionally adjusted for the type of scanner used.

^b^ For this measurement, there were 499 men and 239 women in the intervention group and 482 men and 224 women in the control group.

Table 3 shows multivariable associations between important exposure variables and the lean mass indices. Age had a positive association with all the lean mass indices, which indicated that participants had probably not achieved their peak muscle mass and strength. As expected, there were marked differences in the lean mass indices of men and women. Although socioeconomic position had a positive association with all of the outcome variables, educational level and occupation group were largely unrelated to the assessed outcomes. Dietary energy intake and physical activity level, however, had positive associations with LBM, ASM, and grip strength (energy intake only). Compared with participants with energy intakes in the lowest tertile, participants with energy intakes in the middle and the uppermost tertiles had a higher LBM (approximately 1.2 kg and 2.65 kg, respectively). Energy-adjusted protein intakes were not associated with any of the lean mass indices.

**Table 3.** Multiple Regression Analyses to Examine the Associations of Outcome Variables With Various Determinants in the Follow-up Study of Andhra Pradesh Children and Parents Study Birth Cohort, Hyderabad, India, 2009–2010

	LBM, kg (*n* = 1,375)			ASM, kg (*n* = 1,375)			Grip strength, kg (*n* = 1,435)		
	β	95% CI	*P* Value	β	95% CI	*P* Value	β	95% CI	*P* Value^a^
Age, years	0.43	0.21, 0.65	<0.01	0.12	0.02, 0.22	0.02	0.40	0.20, 0.61	<0.01
Sex									
Male	0	Referent		0	Referent		0	Referent	
Female	−12.04	−12.69, −11.39	<0.01	−6.59	−6.90, −6.28	<0.01	−11.29	−11.91, −10.67	<0.01
Standard of living index^b^									
Tertile 1	0	Referent		0	Referent		0	Referent	
Tertile 2	0.30	−0.32, 0.91		0.11	−0.20, 0.41		0.22	−0.48, 0.93	
Tertile 3	1.69	1.09, 2.29	<0.01	0.74	0.45, 1.02	<0.01	1.16	0.49, 1.83	<0.01
Educational level									
≤Primary school	0	Referent		0	Referent		0	Referent	
Secondary school	0.07	−0.64, 0.77		0.10	−0.25, 0.46		1.03	0.09, 1.98	
College	0.78	−1.10, 2.66	0.78	0.32	−0.44, 1.09	0.45	1.05	−0.49, 2.63	0.07
Occupation									
No paid employment	0	Referent		0	Referent		0	Referent	
Manual work (unskilled/ skilled)	0.34	−0.34, 1.02		0.08	−0.24, 0.42		0.51	0.01, 1.02	
Professional	−0.05	−1.17, 1.28	0.33	−0.22	−0.95, 0.37	0.38	1.05	−0.50, 2.60	0.32
Physical activity^c^									
Tertile 1	0	Referent		0	Referent		0	Referent	
Tertile 2	0.10	−0.63, 0.84		0.10	−0.27, 0.47		0.06	−0.75, 0.89	
Tertile 3	0.71	0.03, 1.39	<0.01	0.45	0.12, 0.77	<0.01	0.14	−0.67, 0.94	0.45
Dietary energy intake^d^									
Tertile 1	0	Referent		0	Referent		0	Referent	
Tertile 2	1.20	0.53, 1.86		0.59	0.25, 0.94		0.81	0.22, 1.40	
Tertile 3	2.65	0.53, 1.86	<0.01	1.26	0.80, 1.71	<0.01	2.17	1.37, 2.96	<0.01
Energy- adjusted protein intake^e^									
Tertile 1	0	Referent		0	Referent		0	Referent	
Tertile 2	0.40	−0.30, 1.11		0.23	−0.15, 0.62		−0.05	−0.85, 0.74	
Tertile 3	0.23	−0.48, 0.94	0.75	0.07	−0.30, 0.44	0.71	−0.36	−1.11, 0.38	0.35
*R*^2^	0.63			0.67			0.56		

Abbreviations: ASM, appendicular skeletal muscle mass; CI, confidence interval; LBM, lean body mass.

^a^ Associations of individual predictors with the LBM indices were examined using multivariable linear regression after adjustment for all the other predictors and nutrition supplementation. *P* values are based on the robust standard errors to account for clustering by village and sibling pair. *P* values for trend are reported for categorical variables (tertiles of standard of living index, physical activity, energy intake, energy-adjusted protein intake, educational level, and occupation). All the *P* values were 2-sided.

^b^ Standard of living index tertiles: <17, 17–20, and >20.

^c^ Physical activity (MET-hour/day) tertiles: <35.3, 35.3–40.7, and >40.7.

^d^ Energy intake (kcal/day) tertiles: <2,273; 2,274–3,316; and >3,316.

^e^ Energy-adjusted protein intake (g/day) tertiles: <69.3, 69.3–73.5, and >73.5.

Table 4 shows the differences in LBM, ASM, and grip strength in relation to the nutrition supplementation after adjustment for the relevant potential confounders. Model 1, which was adjusted for physiological variables (age and sex), indicated lower ASM (β = −0.40, 95% confidence interval: −0.75, −0.05 *P* = 0.02) in the intervention group, with a similar trend in LBM (β = −0.66, 95% confidence interval: −1.36, 0.04; *P* = 0.06); however, there was no difference in grip strength between the 2 groups. After adjustment for socioeconomic and lifestyle predictors in model 2, differences in the 2 groups were slightly higher, with lower values of all the lean mass indices in the intervention group. The magnitude of these differences in all of the outcome variables in relation to early nutrition supplementation was, however, small (<0.1 standard deviation). Additional adjustment for height (model 3) resulted in only a slight reduction in differences in the outcome variables in relation to supplementation.

**Table 4.** Multivariable Association Between Supplemental Nutrition and Lean Body Mass Indicators in Participants Included in the Follow-up Study of the Andhra Pradesh Children and Parents Study Birth Cohort, Hyderabad, India, 2009–2010

	Model 1^a^ (*n* = 1,388)			Model 2^b^ (*n* = 1,375)			Model 3^c^ (*n* = 1,375)		
	β^d^	95% CI	*P* Value^e^	β^d^	95% CI	*P* Value^e^	β^d^	95% CI	*P* Value^e^
LBM, kg	−0.66	−1.36, 0.04	0.06	−0.75	−1.41, −0.09	0.03	−0.64	−1.20, −0.08	0.03
*R*^2^	0.60			0.64			0.75		
ASM, kg	−0.40	−0.75, −0.05	0.02	−0.50	−0.82, −0.12	<0.01	−0.41	−0.69, −0.13	<0.01
*R*^2^	0.65			0.67			0.80		
Grip strength, kg^f^	−0.50	−1.22, 0.22	0.17	−0.81	−1.41, −0.21	<0.01	−0.70	−1.27, −0.12	0.02
*R*^2^	0.54			0.56			0.60		

Abbreviations: ASM, appendicular skeletal muscle mass; CI, confidence interval; LBM, lean body mass.

^a^ Model 1 was adjusted for age and sex.

^b^ Model 2 was adjusted for variables in model 1 and educational level, occupation, tertile of standard of living index (<17, 17–20, and >20), village urbanization (village population <2,000; 2,000–5,000; or >5,000), tertiles of physical activity (<35.3, 35.3–40.7, and >40.7), tertiles of dietary energy (kcal/day: <2,273; 2,274–3,316; and >3,316), and tertiles of energy-adjusted protein intake (<69.3, 69.3–73.5, and >73.5).

^c^ Model 3 was adjusted for variables in model 2 and height.

^d^ β coefficients are the differences (intervention − control) in the outcome variables.

^e^ *P* values (2-sided) were based on linear regression models with robust standard errors to account for clustering by village and household (sibling pair). Additional adjustment for the type of dual energy x-ray absorptiometry scanner was done in case of total lean body mass and ASM.

^f^ The sample size for grip strength in model 1 was 1,447, in model 2 was 1,435, and in model 3 was 1,434.

## DISCUSSION

In the present cohort of rural young adults, exposure to early nutrition supplementation did not have a positive association with adult LBM and muscle strength as we had hypothesized. On the other hand, lifestyle factors, including dietary energy intake, physical activity level, and socioeconomic position, were important determinants of lean mass indices.

A number of studies based on long-term follow-up of birth cohorts from high- and low-income countries have shown a positive relationship between birth weight (an indirect indicator of early nutrition) and adult LBM and muscle strength (4, 6, 28, 29). Conversely, follow-up studies of nutrition intervention trials in pregnant women have shown an inconsistent relationship between early nutrition exposure and LBM of the offspring (9, 10, 30). A widely cited study from Guatemala showed a positive association of a high-energy, high-protein supplement provided to pregnant women and young children with the LBM of the offspring (girls only) during adolescence (9). However, another cluster-randomized trial from the Gambia, which compared protein-energy supplementation during pregnancy (from 20 weeks of gestation to term) (intervention) with that offered during the postpartum period (control), suggested no effect of the intervention on offspring body composition compared with the control group (10). Our study also showed that nutrition supplementation in early life, provided through a government program, did not have a lasting influence on the lean mass indices of the offspring.

Differences between the results of our study and those of the Guatemalan study may be partly related to differences in the study design and the effective supplemental dose. The Guatemalan study was a randomized controlled trial with supervised nutrition supplementation, whereas exposure in our study was ecological, as participants born in the intervention villages were considered to be exposed to the supplement. In addition, differences in the ages at follow-up may have influenced the outcome. Previous follow-up assessment of our cohort members during adolescence showed an indirect positive association of supplementation with LBM (taller height), which is qualitatively similar to the findings in the Guatemalan study (15). This beneficial association between early nutrition exposure and LBM probably did not persist beyond adolescence because of “dilution” of the programming effect of early nutrition by diet and other lifestyle changes over the years.

The negative association of early nutrition supplementation with the adult lean mass indices in our study, although difficult to explain, could be related to a confounding effect of imperfectly measured or unmeasured confounders on the observed relationship. Studies based on long-term follow-up of birth cohorts in transitioning communities are faced with challenges in dealing with complex confounding influences related to rapid socioeconomic and lifestyle changes. The direction of bias resulting from such confounding effect may be either towards or away from the null, depending on the correlation structure and the distribution of confounders in the 2 study groups (31). Despite our efforts to measure the possible confounders with rigorous quality control, inherent inaccuracies in the measurement of some of the exposures (e.g., socioeconomic position, urbanization, dietary intakes, physical activity level) may have resulted in bias because of inadequate adjustment for these confounders (32). Moreover, the effect size of the negative association between early nutrition and later lean mass indices was small (<0.1 standard deviation), and the possibility of this being a chance finding cannot be ruled out (33).

The positive relationship of lifestyle determinants, including dietary energy intake and physical activity level, with the LBM indicators observed in the present study is largely consistent with the existing evidence (34, 35). However, energy-adjusted protein intake was unrelated to the lean mass indicators, unlike in some previous studies that showed a beneficial association of protein intake with lean body mass and muscle mass (36, 37). This could be attributed to the differences in the protein quality between studies: In the previous studies, protein intake was largely from animal sources, whereas in the present study, it was from cereal. Socioeconomic position had a significant positive influence on the LBM indicators, probably because of its positive relationship with the energy intakes.

The major strength of the present study is the comprehensive assessment of early and later-life influences on LBM and muscle strength. The majority of the previous studies, on other hand, have examined these influences in isolation. In addition, nutrition intervention in the present study mimics the real-life situation, as the intervention was a part of an ongoing nutrition program in India (rather than being a controlled nutrition supplementation trial) and therefore allows realistic estimation of the long-term impact of the program. Other strengths include the use of a precise technique for body composition assessment and a large sample size.

The study also has some important limitations that need to be acknowledged. Major limitations include nonrandomization of villages in the baseline study and losses to follow-up. Losses to follow-up were, however, similar in the intervention and control groups, and distributions of birth weight (in a subsample) and sociodemographic characteristics of the participants and nonparticipants among the eligible cohort members were similar in the 2 groups. These losses are therefore likely to be nonsystematic and are less likely to bias the results of the study. Another limitation is the nonavailability of precise estimates of rates of adherence to the intervention. However, indirect evidence of a higher birth weight in the intervention group, as well as information available from the original trial, suggests that a high rate of supplement intake was likely (14). Finally, despite relative automation of the major outcome measure (LBM) used in this study, the possibility of bias arising from the lack of blinding of the fieldworkers cannot be ruled out completely.

In summary, we did not find a long-term positive association between nutrition supplementation in early life provided through a government program and adult LBM and muscle strength. Consistent with existing evidence, current socioeconomic position and lifestyles, including dietary energy intake and physical activity level, were found to be important determinants of the LBM and muscle strength in this setting.

## Appendix

[Table KWT332TB5]

**Appendix Table 1. KWT332TB5:** Characteristics of Young Adults Who Attended and Those Who Did Not Attend Clinics in the Follow-up Study of the Andhra Pradesh Children and Parents Study Birth Cohort, Hyderabad, India, 2009–2010

Characteristic	Intervention Area (*n* = 1,342)					Control Area (*n* = 1,259)				
	Participants (*n* = 738)		Nonparticipants (*n* = 604)		*P* Value^a^	Participants (*n* = 708)		Nonparticipants (*n* = 551)		*P* Value^a^
	Mean (SD)	%	Mean (SD)	%		Mean (SD)	%	Mean (SD)	%	
Age, years^b^	21.7 (1.1)		21.7 (1.1)		0.12	21.7 (1.1)		21.7 (1.1)		0.43
Female sex		32		70	<0.001		31.5		72.4	<0.001
Occupation^c^										
Full-time student		82.5		69.4	<0.001		76.8		61.7	<0.001
Full-time employment		90		19.9			17.8		29.1	
Other (neither, both)		39		10.7			5.4		9.2	
Birth weight, kg^d^	2.72 (0.42)		2.73 (0.38)		0.74	2.64 (0.43)		2.59 (0.43)		0.26

Abbreviation: SD, standard deviation.

^a^ The *P* values are based on unpaired *t* tests or χ^2^ tests for heterogeneity with appropriate degrees of freedom.

^b^ As of January 1, 2010.

^c^ Based on 2003 data. For this measurement, there were 737 participants and 598 nonparticipants in the intervention area and 708 participants and 532 nonparticipants in the control area.

^d^ For this measurement, there were 198 participants and 136 nonparticipants the intervention area and 273 participants and 165 nonparticipants in the control area.
